# Freudian Slip? The Changing Cultural Fortunes of Psychoanalytic Concepts

**DOI:** 10.3389/fpsyg.2019.01489

**Published:** 2019-06-28

**Authors:** Nick Haslam, Lotus Ye

**Affiliations:** Melbourne School of Psychological Sciences, The University of Melbourne, Parkville, VIC, Australia

**Keywords:** culture, culturomics, English, French, psychoanalysis

## Abstract

It is often argued that psychoanalysis has declined in prominence since its ascendance in the mid-20th century. To assess this claim we examined the trajectory of psychoanalytic concepts from 1900 to 2008 in the massive Google Books database. The changing relative frequency of a sample of English-language psychoanalytic terms was explored and compared to a sample of terms in French. The frequency of the English terms was further explored from 2008 to 2017 using the Corpus of Contemporary American English (COCA). The English terms rose steeply from the 1940s and declined steeply from the early 1990s. In contrast, the French terms rose steeply from the 1960s and plateaued from the 1970s. In addition, psychoanalytic terms were markedly more prominent in French since the 1960s. The findings are discussed in the context of historical trends in the reception of psychoanalysis in the Anglophone and Francophone worlds.

## Introduction

Psychoanalysis is often said to be in retreat as an intellectual tradition, a clinical practice, and a cultural phenomenon. Its supposed decline, celebrated by some and mourned by others, has been attributed to a variety of causes. The intellectual merit of psychoanalysis has been repeatedly challenged on scientific and political grounds. Its value as a clinical approach has been undermined on the one hand by the rise of pharmacological treatments and on the other hand by the advent of shorter-term and more “evidence-based” forms of psychotherapy. Its internal frictions have contributed to a growing marginalization and fragmentation of the psychoanalytic community (Stepansky, 2009). Culturally, the influence of psychoanalysis may have lost ground to other perspectives on mind and behavior, such as cognitivism or positive psychology.

Often missing in discussions of the apparent decline of psychoanalytic ideas is the recognition that these ideas may have different trajectories in different cultural contexts. The supposed eclipse of psychoanalysis may only be partial, and largely restricted to the Anglophone world, where many of the fiercest philosophical, psychological, and cultural critiques have been made. Any attempt to assess the changing historical fortunes of psychoanalysis must look beyond the Anglosphere.

The situation in the Francophone world is a case in point. Psychoanalysis came relatively late to France, Freud noting in his 1914 history of the psychoanalytic movement that it was the least receptive to his ideas of the European countries. The first French translation of his work only appeared in 1921 (Mangan, 1950). Despite these late beginnings, psychoanalysis experienced a rapid “blossoming” in the third quarter of the century (Cournut, 1990) and became a touchstone of intellectual life that extended into popular culture and the news media. As Turkle (1992) remarked, in the 1960s “the French attitude toward psychoanalysis swung from denigration and resistance to infatuation” (p.4). Psychoanalytic ideas also became prominent in psychiatry and clinical psychology, Botbol and Gourbil (2018) observing that large proportions of French psychiatrists conduct psychotherapy and describe themselves as psychoanalysts at a time when these proportions are small and diminishing in the United States and the United Kingdom.

Several reasons have been put forward for the continuing prominence of psychoanalysis in Francophone cultures. These include the high value traditionally placed on intellectuals, the tradition of secularism, the emergence of a distinctive French approach to psychoanalysis in the second half of the 20th century, and the centrality of this approach with the cultural revolution of the late 1960s. Whatever its cause, the enduring influence of psychoanalytic ideas in the Francophone world appears to stand in sharp contrast to the decline in the Anglosphere. Indeed, the fate of psychoanalysis in the two worlds appears to be quite distinct, a conclusion supported by a recent analysis showing very low rates of mutual citation of articles published in Anglo-American and French psychoanalytic journals (Potier et al., 2016).

Although the claim that psychoanalytic ideas have had different trajectories of cultural influence in English and French is credible, it has yet to be examined systematically. One way to do so is to use the tools of “culturomics” (Michel et al., 2011) to explore cultural trends by tracking changes in the frequency of words in massive text corpora. Although it lacks the capacity of more qualitative approaches to language analysis to examine detail and complexity, culturomic methods allow historical changes to be precisely quantified using massive text corpora. Using the Google Books corpus, which contains 500 billion words from 5 million digitized books, for instance, changes in the relative frequency of words (as a function of all words) can be examined as an index of their cultural salience. Researchers have used culturomic methods to explore shifts in individualist and collectivist values (Twenge et al., 2012; Hamamura and Xu, 2015; Zeng and Greenfield, 2015), concepts of happiness (Oishi et al., 2013), and concepts of morality (Wheeler et al., 2019), among others.

The present study explored historical changes in the cultural prominence of psychoanalysis by examining shifts in the frequencies of a large set of English and French psychoanalytic terms in two text corpora. The primary corpus was book-based (Google Books) because no comparably large, systematic, multi-lingual, and historically extended language corpus exists, although in principle the analyses could be conducted using newspapers, journal articles, or other sources of text. The English corpus was compared to a French corpus rather than a different language because of the often-remarked difference in the currency of psychoanalytic ideas in the two cultural contexts. (Further research might profitably extend this comparison to additional languages, such as German, Italian, and Spanish.) Contrasts were not attempted within the Anglosphere because widespread joint publication of books in different countries (e.g., the United States and the United Kingdom) obscures any national differences and our primary focus was on cross-linguistic comparison.

This research is not the first to employ a corpus methodology in the psychoanalytic context. For instance, Cariola (2014a, b) has used a computerized dictionary to assess body boundary imagery and to explore psychodynamic themes in *Mein Kampf*, and Salvatore et al. (2017) have used automated text analysis to examine psychotherapy transcripts. However, our study is the first to examine psychoanalytic discourse itself and employs a vastly larger corpus to do so.

The study was descriptive in nature rather than testing hypotheses. Nevertheless, based on historical considerations we anticipated that the trajectories of psychoanalytic terms in English and French would differ in predictable ways. In particular, we expected that English terms would rise in prominence earlier, decline more in recent decades, and be less salient overall in recent decades relative to their French equivalents.

## Materials and Methods

### Dictionary Creation

Prior to conducting the analysis of temporal trends in the prominence of psychoanalytic concepts, we developed a sample (i.e., a “dictionary”) of psychoanalytic terms. The goal of the sampling was not to exhaustively represent the psychoanalytic theoretical tradition but to develop a substantial set of terms distinctive to that tradition. Candidate terms were initially sourced from English-language dictionaries of psychoanalytic concepts (De Mijolla, 2005; Akhtar, 2009), retaining an initial sample of 56 one- or two-word terms that were judged by the authors to be reasonably specific to psychoanalytic theory. French translations of these terms were sourced from one of the dictionaries (De Mijolla, 2005), supplemented by literature searches. Robust translations of four terms (“instinctual drive,” “preoedipal,” “psychosexual theory,” “topographic theory”) could not be located, so the initial French sample contained 52 terms.

### Data Collection and Analysis

Google NGram was used to record the relative frequency of each term (as a proportion of all terms) in each year from 1900 to 2008, when the Google Books corpus concludes. All searches used the English or French book corpora in Google NGram and were case sensitive (with only normally capitalized words capitalized). They generated unsmoothed time series of the relative frequencies of each term for the 1900–2008 periods. As in Wheeler et al. (2019), these time series were rescaled so that the year with the highest relative frequency was scaled as 100 and all other years were represented as percentages of that peak frequency. To test whether any terms were not sufficiently distinctive to the psychoanalytic tradition, we excluded terms whose mean scaled frequency from 1900–1909 was greater than 10 (i.e., they were widely used, reaching >10% of their eventual peak frequency, before psychoanalysis became prominent). This resulted in the exclusion of “the Ego” from the English sample and “association libre,” “le Moi,” “le Ça,” and “l’Inconscient” from the French sample. To examine the trajectory of English psychoanalytic concepts we therefore retained 55 terms (56−1) and to examine the French trajectory we retained 48 (52−4; see Supplementary Appendix). Where direct comparison between languages was required we restricted analysis to the English and French versions of these 48 shared terms. Analyses of historical trends in the use of psychoanalytic terms were conducted in two ways: by taking average scaled values across the terms and by summing the relative frequencies of all terms. The first method gives each term equal weight regardless of its frequency, whereas the second better summarizes the aggregate use of psychoanalytic concepts by giving greater weight to more frequently used terms.

Analyses indicated a trend in the last two decades of the English Google Books corpus so we conducted additional data collection to extend the study period beyond 2008. For this purpose, we used the Corpus of Contemporary American English (COCA; Davies, 2010), which contains 20 million words annually from 1990 to 2017, sampled approximately equally from spoken language, fiction, popular magazines, newspapers, and academic journals. Although COCA differs from Google Books in using multiple genres beyond books and in being sourced from the United States rather than all Anglophone countries, we judged it the best available corpus for assessing whether the trend continued.

## Results

**FIGURE 1:**
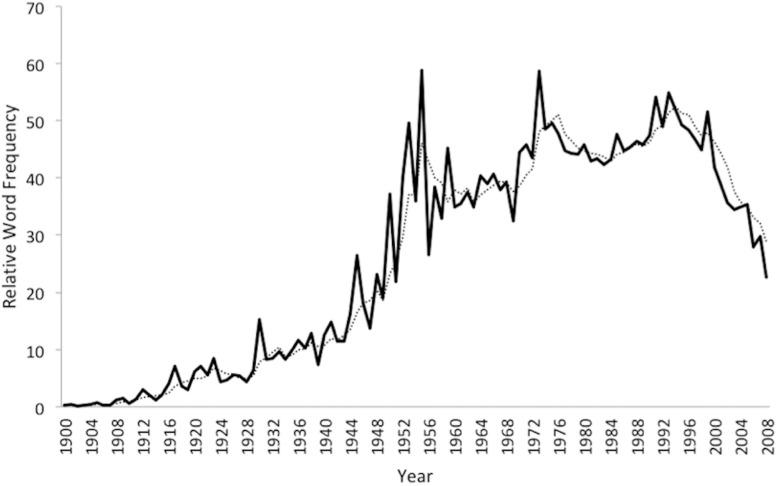
Scaled Relative Frequency of 55 English Psychoanalytic Terms (Google Ngram), 1900–2008.

Figure 1 presents the mean scaled relative frequency of the set of 55 English psychoanalytic terms (the plot of the 48 shared terms was extremely similar). The terms rise most steeply in relative frequency in the 1940s and 1950s, reaching a peak in the mid-1950s followed by a second peak in the mid-1970s. A third peak appears in the early-1990s, after which there is a steep decline: from 1993 to 2008 the mean scaled relative frequency drops 58.8%.

**FIGURE 2:**
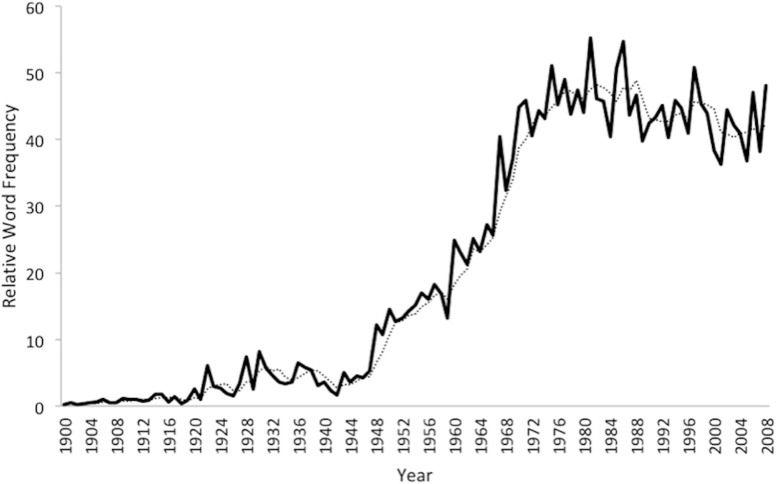
Scaled Relative Frequency of 48 French Psychoanalytic Terms (Google Ngram), 1900–2008.

Figure 2 shows the comparable time series for the 48 French terms. The steepest rise in relative frequency occurs in the 1960s, one or two decades after the English rise, and there is no meaningful decline once the relative frequency reaches a plateau in the mid-1970s. Whereas the most common decade in which the French terms reached their peak relative frequency was the 1980s (median = 1978), a majority of the 48 equivalent English terms reached their peak in the 1950s (median = 1956).

**FIGURE 3:**
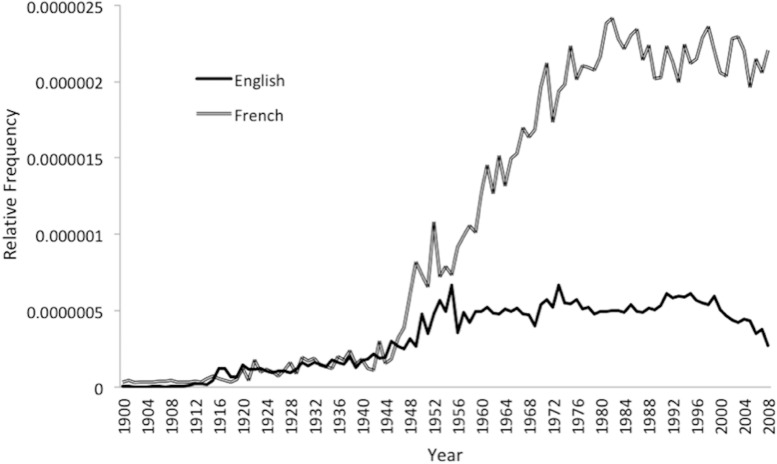
Summed Relative Frequency of 48 English and French Psychoanalytic Terms (Google Ngram), 1900–2008.

The analyses reported in Figures 1, 2 relate to scaled relative frequencies, which give equal weight to each term regardless of its frequency of use. Figure 3 presents the summed relative frequencies of the shared 48 terms in English and French, showing the overall frequency of each set of terms. The time series show very similar shapes to Figures 1, 2, with the French series plateauing from the mid-1970s and the English series dropping 55.3% from its 1990s peak to 2008. However, the psychoanalytic terms have a much higher aggregate relative frequency in French than in English, implying a much higher level of cultural prominence. In 2008, the terms were collectively more than eight times as frequent in the French Google Books corpus than in the English corpus.

**FIGURE 4:**
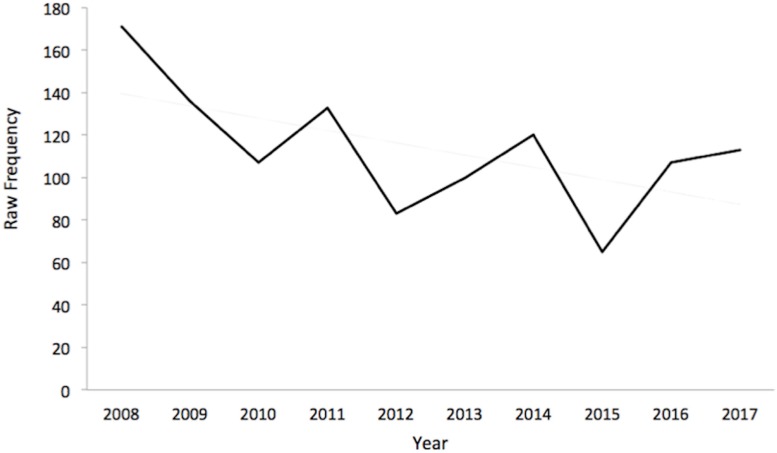
Summed Frequency of 55 English Psychoanalytic Terms Corpus of Contemporary American English (COCA), 2008–2017.

To examine whether the decline in the relative frequency of psychoanalytic terms in English continued after 2008, the aggregate frequency of the 48 shared terms (out of the ∼20 million words in each year) was determined using COCA from 2008 to 2017. Figure 4 shows that COCA indicates a declining trend (*r* = −0.60, *p* = −0.07). Over the 10-year period, the trendline estimates a 37.8% decrease in the frequency with which the psychoanalytic terms appeared. If this decrease is combined with the 55.3% decline from 1995 to 2008 shown in Figure 4 it would imply an overall decline of 70.4% from 1995 to 2017.

## Discussion

As we anticipated, the historical trajectories of psychoanalytic terms differed appreciably in English and French. Within the Google Books corpus, English terms rose in relative frequency much earlier than French terms, indicating an earlier cultural incorporation of psychoanalytic ideas in the Anglophone world. The trajectory of the English terms also showed a steep decline in the past quarter century that was not matched by a comparable decline among the French terms. Although the declining influence of psychoanalysis in the Anglosphere had been announced on many earlier occasions, it appears to have commenced in the early 1990s. Finally, our findings support the claim that psychoanalytic ideas retain a stronger influence in the Francophone world and quantify the stark difference in that relative influence.

Our findings paint a nuanced picture of the changing cultural prominence of psychoanalytic concepts in the Anglophone world over the past century. While a supposed decline of psychoanalytic ideas often claimed by critics during the 1960s and 1970s, our findings show that a small early decline from the mid-1950s was followed by several rebounds prior to the larger decline observed since the early 1990s. Although these rebounds point to the resilience of psychoanalytic ideas within Anglophone cultures of the late 20th century, the drop over the past quarter-century has been marked. Factors contributing to it probably include the growth of alternative therapeutic frameworks in the mental health disciplines, including the rise of psychopharmacology (Baldessarini, 2014) and cognitive-behavior therapy, and the turn toward a medicalized approach to diagnosis and classification ushered in by the DSM-III in 1980 (Blashfield et al., 2014). Psychoanalytic ideas remain influential within many academic contexts in the English-speaking world, and especially in the humanities, but our findings suggest that their currency outside these contexts has diminished.

In contrast to our English language findings, our analysis of French psychoanalytic terms demonstrates no downward trajectory and a much higher recent elevation after a delayed climb. This later reception of psychoanalytic ideas may be due in part to two factors in post-WWII France: the resistance of existentialist thinkers to them and the exodus of many European analysts to the United States. Despite this delay, the stable high relative frequency of French terms since the 1970s indicates that psychoanalysis remains a culturally prominent force in the Francophone world. Indeed, while recognizing the diversity within the Francophone world (e.g., in Belgium, Canada, and Switzerland), the key influence of psychoanalysis in France has been noted in a variety of disciplines that range from psychology and medicine to politics and sociology (Botbol and Gourbil, 2018). The sustained prominence of psychoanalytic ideas in France may in part be due to the intertwining of French psychoanalytic and philosophical thought, which stands in contrast to the traditional separation in the Anglophone world (Birksted-Breen et al., 2010). The incorporation of psychoanalytic thought into the highly influential French intellectual movements of the 1960s and since may be a key contributor to its ongoing cultural vitality. In addition to the greater embedding of psychoanalytic ideas within broad intellectual trends within the Francophone world, psychodynamic theories also play a larger role in the clinical practice of psychiatrists and psychologists there. As several writers have noted, the DSM has been markedly less popular in the French-speaking world than in United States, and cognitive-behavioral approaches to psychotherapy have been adopted at a much slower pace (Amouroux, 2017).

Although our findings reveal trends in the salience of psychoanalytic ideas in two cultural contexts, caution should be applied in making inferences regarding the trajectory of psychoanalysis in a broader sense. First, our sample of terms was weighted toward classic psychoanalytic concepts, and a sample of terms coined in more contemporary psychoanalytic writing might not show the same trajectories. Second, our findings also do not reveal the full cultural influence of psychoanalytic ideas, as this influence may have been expressed by incorporating or co-opting these ideas without retaining of distinctively psychoanalytic terminology (e.g., attachment theory). Third, our findings speak only to the changing cultural prominence of psychoanalytic concepts as revealed in published books, not to the changing or unchanging value or validity of those concepts. Finally, our findings relate to the salience of psychoanalytic concepts in the culture at large, as revealed in a vast and very broad sample of its books and have little to say about the state of publishing within the specialist field of psychoanalysis itself (cf. Stepansky, 2009), which represents a very small fraction of that sample. However, the general decline in the salience of psychoanalytic concepts in the Anglosphere might be associated with a comparable decline in the volume of psychoanalytic books being published. If psychoanalytic publishing is also in decline or is not growing at the same rate as publishing in other fields, that might reflect or contribute to the broader decline in the prominence of psychoanalytic ideas. Further research might clarify these matters.

On Sigmund Freud’s death in 1939, W. H. Auden wrote that he “is no more a person now but a whole climate of opinion.” Our findings indicate that in the Anglosphere this climate was just starting to heat up and would rise steeply for the next two decades, before cooling again in the 1990s and since. Cultural climate change followed a different course in the French-speaking world, heating up later and remaining warm thereafter. Whatever changes may be in store for the cultural prominence of psychoanalytic ideas, it is important to recognize that these changes may vary in different cultural settings.

## Data Availability

The raw data supporting the conclusions of this manuscript will be made available by the authors, without undue reservation, to any qualified researcher.

## Author Contributions

LY collected the data. Both authors conceptualized the study, conducted data analyses, drafted the manuscript, and read and approved the submitted version.

## Conflict of Interest Statement

The authors declare that the research was conducted in the absence of any commercial or financial relationships that could be construed as a potential conflict of interest.
