# Supplementary material for: Freudian Slip? The Changing Cultural Fortunes of Psychoanalytic Concepts
Source: Front Psychol. 2019 Jun 28;10:1489. doi: 10.3389/fpsyg.2019.01489 (PMC6611072; doi:10.3389/fpsyg.2019.01489)
Supplement: Supplementary file 1 [file Table_1.DOCX]

**Appendix**

Terms used in both languages (*n*=48): abreaction/abreaction; anal character/caractère anal; anal stage/stade anal; countercathexis/contre-investissement; castration anxiety/angoisse de castration; castration complex/complexe de castration; cathexis/investissement; countertransference/contre-transfert; death instinct/pulsion de mort; defense mechanism/mécanisme de défense; Ego Ideal/ideál du Moi; Freudian/Freudienne; genitality/génitalité; genital stage/stade genital; introjection/introjection; latency stage/période de latence; latent content/contenu latent; libido/libido; life instinct/pulsion de vie; manifest content/contenu manifeste; negative therapeutic reaction/réaction thérapeutique negative; object relations/relations d'objet; Oedipal complex/complexe d'Oedipe; oedipal/oedipien; oral character/caractère oral; oral stage/stade oral; overdetermination/surdétermination; parapraxis/acte manqué; penis envy/envie du pénis; phallic character/caractère phallique; phallic stage/stade phallique; pleasure principle/principe de plaisir; polymorphous perversity/pervers polymorphe; the Preconscious/le Préconscient; primal repression/refoulement originaire; psychic apparatus/appareil psychique; psychic reality/réalité psychique; psychoanalysis/psychanalyse; psychoanalyst/psychanalyste; psychoanalytic/psychanalytique; psychosexual stages/stades du développement psychosexual; reaction formation/formation réactionnelle; reality principle/principe de réalité; repetition competition/compulsion de repetition; seduction theory/théorie de la seduction; screen memory/souvenir-écran; Sigmund Freud/Sigmund Freud; the Superego/le Surmoi

Terms used in English only (n=7): free association, instinctual drive, preoedipal, psychosexual theory, the Id, the Unconscious, topographic theory
